# Annotations

**Published:** 1900-04-28

**Authors:** 


### Annotations.

### Advertising.

There are certain clever people who are always striving after altruistic ideals, and when such people happen to be doctors they are subject to outbreaks of indignation at finding the pure stream of science and ethics, which ought, as they think, alone to flow from the fount of medical journalism, sullied by advertisements. What, they would say, has a noble profession to do with advertising, and with all the push and tumble of this self-seeking world? All of which is very charming. Perhaps, if they would look at the balance-sheet of the British Medical Association, which was published last week, they might gather some little idea of the extent to which science and business are interlocked and depend mutually upon one another. From this balance-sheet it appears that the cost of producing the *British Medical Journal* was, roughly, half as much again as all the subscriptions put together, and that all the profits, together with the expenses of running the Association, of the committees, of the scientific grants, the scholarships, and all the rest of it, came out of the patent foods, the coated pills, the meats, and malts, and milks against the advertisement of which the purists now and again raise their voices.

### The Office of Coroner.

A little time ago the Coroners' Society of England and Wales passed the following resolution: "That it is the opinion of the council that in cases of death from violence, or from any unnatural cause, it is a common law duty imposed upon the medical practitioner in attendance, among others, to report to the coroner," and in a covering letter in which this resolution was forwarded to us this supposed duty was considerably enlarged. Without entering upon the very obvious criticisms which must arise in regard to this somewhat naive suggestion, we may say at once that the medical profession at large has by no means jumped at it. Some even have held to the not entirely inexcusable notion that it is the coroner's duty, not theirs, to ascertain—with assistance of his jury—whether a death did or did not arise "from violence or from any unnatural cause," and that if they honestly give their evidence when called upon they do all that society can require of them. Meanwhile it seems not unfair to ask how far coroners are necessary under present conditions of society, and, if necessary, how far they do what is wanted of them. There are many who entirely disbelieve in the utility of the coroner's office, considering that the system of police, by the ramifications of which the whole country is covered, is amply sufficient to afford all the safeguards which can be given to the community by the more antiquated arrangement. They hold also that the manner in which coroners' inquests are often conducted tends to bring the law into contempt; that in practice no action is taken except through the police, who might as well deal with the cases from the beginning; that the coroner's officer is a go-between who has in many cases usurped a position which does not make for justice; and that the unbridled freedom of tongue which some coroners allow both to themselves and their juries is not only a tyranny and an impertinence, but is often the cause of much undeserved and irremediable injury to innocent people. There can be no doubt that a substantial basis for all these objections to the coroner's court as it now is could easily be brought forward if it were necessary so to do. Still, such scandals need not be. They are not essential, and there is no reason why the coroner's office should not be purged of its defects and made again to fill a proper place in the British constitution. Sooner or later a time must come when a means must be provided for a complete registration of all deaths, and for making full inquiry into the cause of death in every case which cannot be properly met by a medical certificate, and the officers (medical officers for preference) who will have the superintendence of this department, taking up as they would the work of the coroners, might as well take their title also. But the organisation must be very different from the effete arrangements now in vogue, and must be intimately in touch with the other great State departments, on the one hand with the police and on the other with the Registrar-General. We must get rid of the absurdity of the same occurrence being investigated both by the police and by various coroners, and we must get rid of the condition of affairs which is brought to light in the last annual report of the Registrar-General, who shows that in 14 per cent. of all inquest cases the returns of the cause of death, the very thing the coroner is appointed to inquire into, were so indefinite as to be worthless for scientific purposes.

### Dr. Glover and his Constituents.

Dr. Glover is to be congratulated on having at once repudiated the view that a direct representative of the profession on the General Medical Council is bound to vote according to any mandate he receives from his constituents, or from people professing to speak in their name. Dr. Toogood had written to him in reference to the Midwives Bill, saying, "Whatever your personal views, we claim that you know the mind of your constituency, and occupy your seat on the General Medical Council for the purpose of representing not your own views, but those of your constituents." To this view of his duty Dr. Glover entirely demurs. He not only questions whether the party represented by Dr. Toogood does really represent the mind of his constituents, but he maintains that, even if it did, he is not bound by its behests. He was sent to the Council, he says, by those who well knew his views, to vote not mechanically but after grave deliberation with twenty-nine other gentlemen, also representing—although too indirectly—the profession, the medical authorities, and the State, and he protests against this attempt to reduce the direct representatives to "mere voting machines, to receive the last mandate of any clique of the profession that thought itself a saviour of society and gave its orders to its slaves." Of course, Dr. Glover is right. A direct representative of the medical profession would, we fear, have to be a very silent member if he were not allowed to speak until he knew what his constituents wanted. Do they know it themselves? Take any question, even this rampant one of the Midwives Bill. Who is to find out what the profession really wants at the present time? Certainly no guidance can be got from a little meeting at Charing Cross.

